# Role of Ion Dissociation on DC Conductivity and Silver Nanoparticle Formation in PVA:AgNt Based Polymer Electrolytes: Deep Insights to Ion Transport Mechanism

**DOI:** 10.3390/polym9080338

**Published:** 2017-08-04

**Authors:** Shujahadeen B. Aziz, Ranjdar M. Abdullah, Mariwan A. Rasheed, Hameed M. Ahmed

**Affiliations:** 1 Advanced Polymeric Materials Research Lab., Department of Physics, College of Science, University of Sulaimani, Qlyasan Street, Sulaimani 46001, Iraq; ranjdar.abdullah@univsul.edu.iq (R.M.A.); hameed.ahmad@univsul.edu.iq (H.M.A.); 2 Development Center for Research and Training (DCRT), University of Human Development, Qrga Street, Sulaimani 46001, Iraq; mariwan.rasheed@uhd.edu.iq

**Keywords:** PVA electrolyte, silver nitrate, SPR absorption peak, impedance plots, DC and AC conductivity, activation energy and barrier height

## Abstract

In this study, the role of ion dissociation on formation of silver nanoparticle and DC conductivityin PVA:AgNO_3_ based solid polymer electrolyte has been discussed in detail. Samples of silver ion conducting solid polymer electrolyte were prepared by using solution cast technique. Absorption spectroscopy in the ultraviolet–visible (UV–Vis) spectral region was used to investigate the formation of silver nanoparticles. Broad and sharp peaks due to plasmonic silver nanoparticles subjected to ion dissociation have been observed. The influence of dielectric constant on the intensity of surface plasmonic resonance (SPR) peaks attributed to silver nanoparticles was discussed. From impedance plots, the diameter of high frequency semicircle was found to be decreased with increasing salt concentration. The DC conductivity in relation to the dielectric constant was also explained. From the AC conductivity spectra, the dc conductivity was estimated to be close to that calculated from the bulk resistance. The temperature dependence of the DC conductivity was studied and found to follow Arrhenius equation within two distinguished regions. The AC conductivity at different temperatures has been studied to comprehend the ion conduction mechanism. The AC conductivity against frequency was found to obey the universal power law of Jonscher. Three distinct regions were recognized from the spectra of AC conductivity. The frequency exponent (*S*) was calculated for the dispersive region of the measured AC conductivity spectra. Various models were discussed to explain the behavior of *S* value with temperature. The behavior of *S* value with temperature was then used to interpret the DC conductivity pattern against 1000/T. Finally, from the comparison of calculated activation energy (*E*_a_) and maximum barrier height (*W*_m_), deep insights into ion conduction mechanism could be grasped.

## 1. Introduction

In recent years, there has been a growing interest in the development of solid polymer electrolytes for various electrochemical device applications, such as electrochemical capacitors, fuel cells, solar cells, high energy density cells and gas sensors [1]. Solid polymer electrolyte (SPE) ion conductors are favored over the conventional ionic solids and liquid electrolytes due to their mechanical stability, flexibility, durability and lightweight [2]. The earliest and the most widely studied polymer electrolytes in the literature are polyethylene oxide (PEO) based systems. However, the main drawbacks of using PEO in the preparation of polymer electrolytes are its strong affinity to crystallize and its low ionic conductivity [3]. The literature reviews revealed that most researchers focused on other types of polymers, such as chitosan [4,5,6,7,8,9], methyl cellulose [10,11,12,13,14] and poly(vinyl alcohol) [1,2,3,14,15,16]. Polyvinyl alcohol (PVA) polymer is widely used in electrochemical and electrochromic devices due to its bio-compatibility and superior mechanical properties. PVA has excellent and easy thin-film formation capability, sufficient hydrophilic properties, and a high density of reactive chemical functions that are ideal for cross-linking by chemical and thermal treatments [17]. In addition, PVA possesses a carbon chain backbone structure with hydroxyl (OH) groups connected to methane carbons, which can be a source of hydrogen bonding and thus assist polymer electrolyte (PE) formation [14]. Polymer electrolyte membranes containing silver ions dissolved in a polymeric solvent have paid considerable attention due to their excellent solid state separation performance [18]. The oxygen atoms of a polar polymer are known to reduce silver ions and create silver metal nanoparticles [19]. The reduction of silver ions to silver nanoparticles has been observed through optical micrograph techniques in PEO:AgSCN based solid polymer electrolytes by Sekhon et al. [20]. In our previous studies, the reduction of silver ions to metallic silver nanoparticles in chitosan based solid polymer electrolytes has been investigated [4,21,22]. However, the stability of silver ions in silver–polymer electrolyte membranes is one of the big concerns in electrochemical device application [22].In polar polymers, silver ions reduction to silver nanoparticles has been reported to be an excellent method to prepare polymer composites with small optical band gaps [23]. It is well known that uncommon electrical and optical properties can be exhibited from nanosized metallic materials, which can clearly be different from those of bulk materials [24]. Such singular features of nanosized materials can be useful in many device applications. Yu et al. have utilized PVA:Ag nanocomposites in the application of surface-enhanced Raman spectroscopy [25]. To the extent of our knowledge, the study of SPE based on polyvinyl alcohol incorporated with silver salts is limited. In this study, electrical, optical and morphological characteristics of PVA:AgNO_3_ have been investigated, aiming to understand the role of ion dissociation on the SPR peak and ion transport behavior. Moreover, from the comparison of activation energy with maximum barrier heights (*W*_m_), better understandings of ion transport mechanism can be grasped.

## 2. Experimental Details

### 2.1. Materials and Sample Preparation

The materials used in this study were high molecular weight polyvinyl alcohol (PVA) polymer of 98–99% hydrolyzed (molecular weight, *M*_w_ = 98,000 g/mol) and silver nitrate (AgNO_3_) (molecular weight, *M*_w_= 169.87 g/mol) of 99.99% purity. These raw materials were supplied by Alfa Aesar (Haverhill, MA, USA) and Sigma-Aldrich (St. Louis, MO, USA), respectively. They were used without further treatment or purification. Double-distilled water was used as a solvent to prepare the PVA:AgNO_3_ nanocomposites. In this study, the conventional solution cast technique has been used for the preparation of solid polymer electrolytes based on PVA. First, 1 g of PVA was dissolved in 50 mL distilled water at 80 °C. The mixture was stirred continuously by a magnetic stirrer for several hours until the PVA powder is fully dissolved, obtaining a clear viscose solution. The PVA solution was then left to slowly cool down to room temperature. To these sets of solution, 5–25 wt % of AgNt has been added separately with continuous stirring to obtain homogeneous solutions. After casting into different plastic Petri dishes, the solutions were left to dry at ambient temperature until electroylte films were formed. Finally, for further drying process, the films were kept in desiccators with blue silica gel desiccant. Table 1 shows the prepared PVA based solid polymer electrolytes samples with various AgNO_3_ concentrations.

### 2.2. Characterization Techniques 

The ultraviolet–visible (UV–Vis) absorption spectra of the prepared films were recorded using a Jasco model V-570 (UV–Vis-NIR) spectrophotometer within the wavelength range 180–1800 nm. To study morphological appearance of the samples, a scanning electron micrograph (SEM) image was taken using FEI Quanta 200 FESEM scanning electron microscope (Hillsboro, OR, USA). The microscope was fitted with an energy dispersive X-ray (EDX) analyzer (Oxford Inst., model INCA Energy 200, Abingdon, UK) for elemental chemical analysis of the PVA:AgNt systems. Before observation, the SPE films were attached to aluminum holder using a conductive tape, and then coated with a thin layer of gold. The impedance of the samples was measured in the temperature range 303–363 K and frequency range 50 Hz–1 MHz, using the HIOKI 3531-Z LCR Hi-tester (Nagano, Japan). Here, the films were fixed on a conductivity holder with stainless steel blocking electrodes of diameter 2 cm.

## 3. Results and Discussion

### 3.1. Absorption and Morphological Study

**Figure 1:**
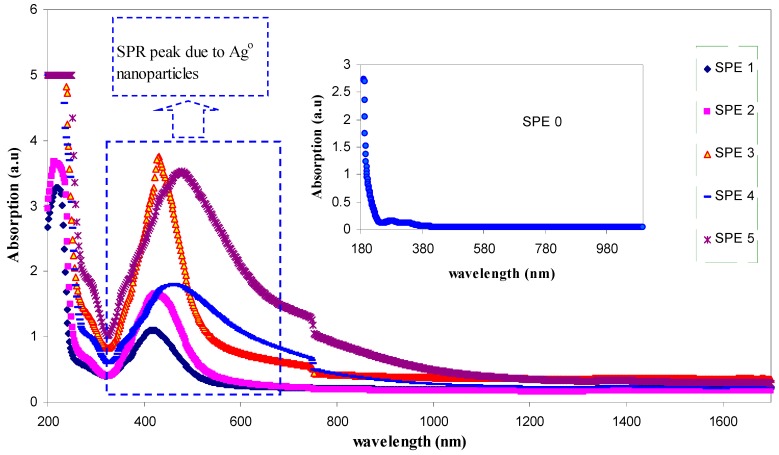
Absorption spectra of pure PVA (inset) and PVA:AgNt solid films. The SPR peak appearing at about 422 nm for PVA:AgNt samples is related to the existence of silver nanoparticles.

**Figure 2:**
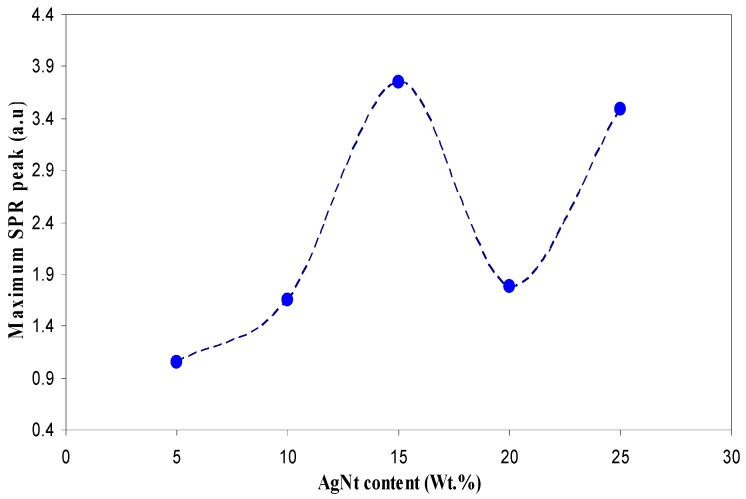
Maximum SPR absorption peak versus salt concentration.

**Figure 3:**
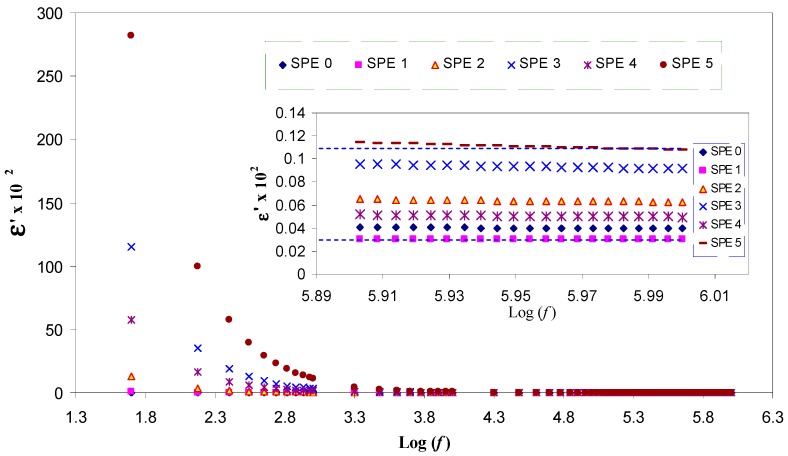
Frequency dependence of dielectric constant for pure PVA and doped PVA samples. The inset shows the dielectric constant for all the samples at high frequencies.

**Figure 4:**
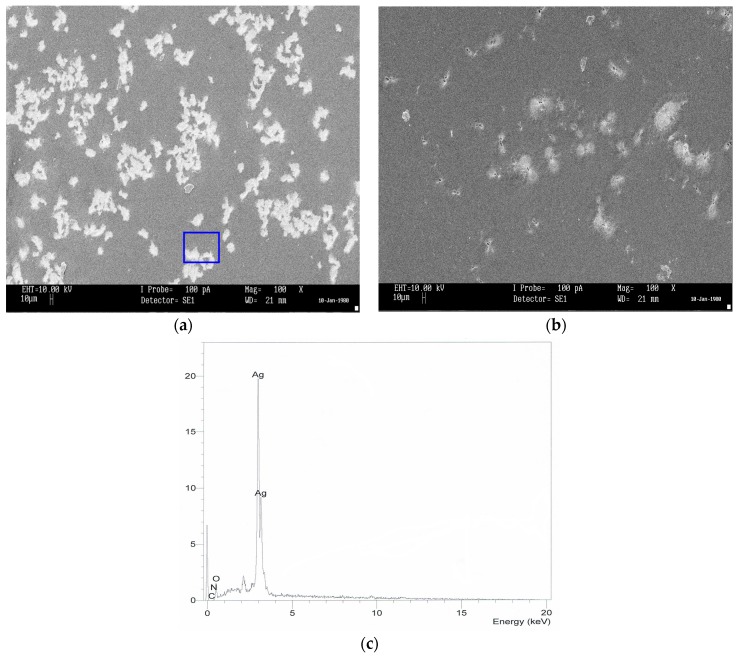
SEM image for: (**a**) SPE2; and (**b**) SPE3; and (**c**) EDX for white specs inside the blue box. The white specs appearing on the film surface are attributable to metallic silver particles.

The UV–Vis absorption spectra are recognized to be quite sensitive to the silver nanoparticles formation [4,26]. Recently, investigation on the optical properties of silver nanoparticles has been performed extensively [27]. It has been reported that silver nanoparticles and their clusters can exhibit a characteristic surface plasmonic resonance (SPR) band in the ultraviolet and visible region, and their heights give information about the concentration of the nanoparticles [4,21,22,28]. Figure 1 shows the absorption spectra for the pure (inset) and doped PVA samples. Here, distinguishable SPR peaks have been observed in the absorption spectra for each of the doped samples. The SPR phenomenon is related to the collective oscillation of the electrons in the valence band in response to the incident beam (i.e., plasmon excitations). The SPR peak appears when the frequency of the incident photons equal to the natural frequency of surface electrons oscillating against the restoring force of their positive nuclei [4,21,29]. It is clear that the intensity of peak maximum increases with increasing AgNt concentration. It is obvious in Figure 2 that the peak maximum has been decreased at 20 wt % of AgNt and then increased for higher AgNt (25 wt %) concentrations. The possible reason for this result might be deduced from ion association phenomenon, which is commonly occurred in polymer electrolytes. This phenomenon will be discussed in detail later in electrical characterization section. It is well confirmed that the plasmon resonance depends strongly on the dielectric constant of the ambient media, a property which is broadly utilized in plasmonic sensors [21,30,31]. Figure 3 shows the dielectric constant values of all the samples as a function of frequency. It can be seen that the dielectric constant increases with increasing the AgNt concentration. One can see that, in Figure 3, the dielectric constant appears to be small for the AgNt concentration of 20 wt % and then becomes large for concentration of 25 wt %. Such result indicates that the measurement of the dielectric constant is an informative test to investigate the SPR phenomena. The broad absorption peaks observing at low salt concentration can be attributed to the wide range of particle size distribution [32]. Figure 4a shows an SEM backscatter electron image of the morphology of SPE2 sample. It is clear from the SEM image that particles appeared as white spots on the polymer surface with diverse sizes. Such wide distribution of the particle size is responsible for the peak broadening of the SPE2 sample. Similar behavior has been reported by Alqudami et al. [32]. However, the SEM image taken for SPE3 sample shows a smaller number of Ag^0^ particles, as shown in Figure 4b. Thus, the sharp behavior of the absorption spectra of SPE3 sample shown in Figure 1 can be related to the non-distribution of silver particles. The EDX spectrum of the particles shown in the blue box of Figure 4a identifies the presence of metallic silver particles through two peaks. Previous studies confirmed that sharp intense peaks of Ag^0^ particles at energy ranges between 3 and 3.6 eV imply a large number of silver nanoparticles [22,23,32]. The intensity of SPR peaks obtained in this study is higher than those reported in earlier studies [25,32]. It is well stated that the incorporation of nanosized metal particles into polymer matrices is of great interest for many purposes [32]. Yu et al. used PVA:Ag^0^ nanocomposites for surface-enhanced Raman scattering [25]. The stabilized nanoparticles can also be considered in their catalysis, magnetic, optical, mechanical and electrical properties [32]. 

### 3.2. Conductivity Study at Room Temperature

The DC conductivity and dielectric constant measured for all the samples at room temperature are presented in Table 2. It is clear that the DC ionic conductivity for the PVA:AgNt based electrolytes has been significantly increased by three orders of magnitude with the addition of different amount of AgNt concentration. Such increase in the DC conductivity can be attributed to the increase of mobile ions. The increase in charge carrier concentration would lead to an increase in the DC conductivity, which can be more understood through the following general expression for DC conductivity at ambient temperature [5],

σ = *Σn*_i_·*q*_i_·µ_i_ (1)

where *n*_i_ indicates the charge carrier density, *q* is 1.6 × 10^−19^C, and µ_i_ is the mobility of the ions. The DC conductivities for the samples have been estimated from the bulk resistance (*R*_b_), which can be obtained from the intersection of real parts of the impedance plots. It can be seen in Table 2 that the DC conductivity and the dielectric constant have been dropped at the AgNt concentration of 20 wt %. Although many polymer electrolytes with excellent conduction properties have been developed, there is still a need to fully understand their ion transport mechanism [33,34]. Previous studies have found that there exist three different ionic constituents in polymer electrolytes, depending on the ion concentration, namely free ions, contact ion pairs and ion aggregates [15,19]. In this study, the decrease in DC conductivity at 20 wt % of AgNt can clearly be attributed to the decrease in free ions, owing to the formation of ion–ion pairing. This finding is similar to the results reported by other researchers, who studied various polymer electrolyte systems using the ion association concept and charge multiples formation [15,35]. In addition, we confirmed in our previous works [5,7,35,36] that dielectric constant has a strong relationship with the DC ionic conductivity. The relationship between dielectric constant and concentration of charge carrier given by *n = n_o_*exp*^(−U/^*^ε’*KT)*^, where *U* refers to dissociation energy and ε’ is the dielectric constant, has been well established in literatures [5,35,36]. The decrease in dielectric constant is an indicator of decrease in carrier concentration and therefore a drop in the DC conductivity can be expected. The dielectric constant values presented in Table 2 have been estimated by extrapolating the data in the plateau regions to the Y-axis (see the inset of Figure 3). Similar trend of ε’ with salt concentration reveals that the conductivity behavior of polymer electrolytes can be understood from the dielectric study. It is clear from Table 2 that at 25 wt % of AgNt the DC conductivity increased again. This related to the fact that more salts are added and thus a high dielectric constant and consequently according to Equation (1), a higher DC conductivity resulted. The maximum DC conductivity measured for the current study is found to be 4.5 × 10^−6^ S/cm and greater than those reported for PVA:Mg(NO_3_)_2_ and PVA:KCl, which are 7.36 × 10^−7^ S/cm and 9.68 × 10^−7^ S/cm, respectively [1,15]. However, it is very close to the obtained value reported by Sheha et al. [14], in which the highest conductivity was 9.8 × 10^−6^ S/cm for PVA, PEDOT:PSS-MgBr_2_ system.

**Figure 5:**
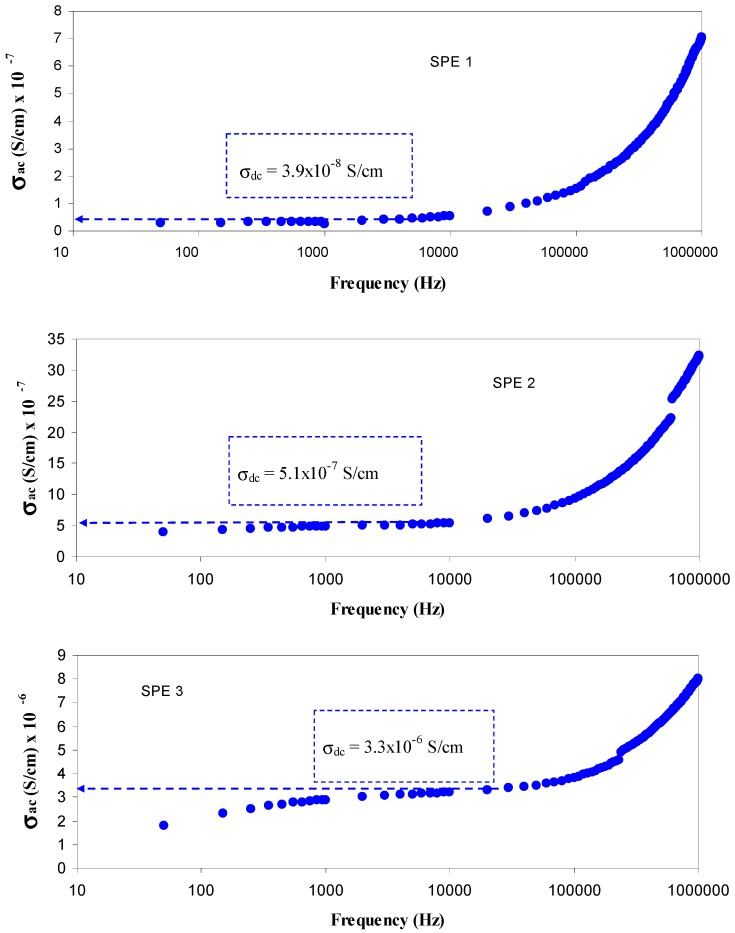

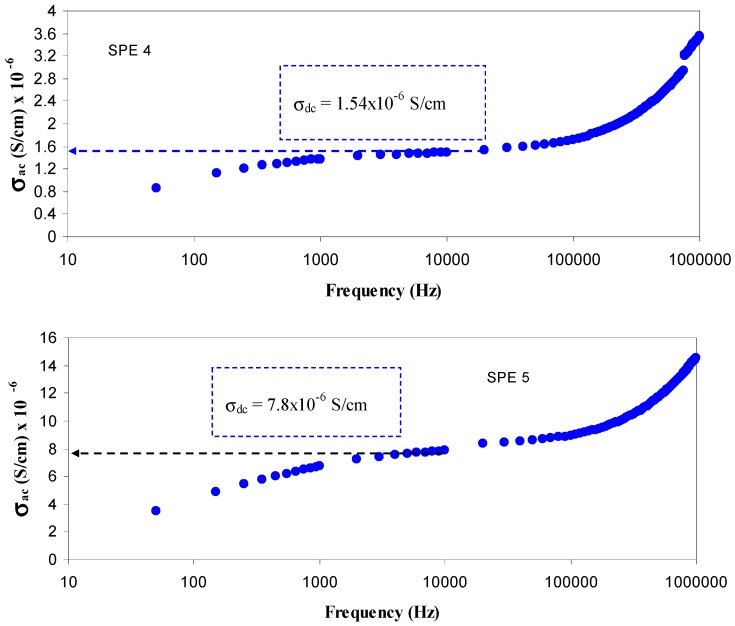
AC conductivity versus frequency for PVA:AgNt (SPE1 to SPE5) samples. The tail observed at low frequencies are ascribed to electrode polarization and the frequency independent plateau region of the conductivity is related to the DC conductivity and shifted to higher frequency side with increasing salt concentration.

A complete understanding of the mechanism of charge transport in the polymer electrolytes is very important from both fundamental and technological perspectives [37]. Previous studies have confirmed that the plateau region of AC conductivity can be used to estimate the bulk DC conductivity [5,36,37]. This is related to the fact that impedance measurement is one of the powerful mean to characterize and understand the charge transport mechanism in complex materials [37]. Obviously, the AC conductivity spectra shown in Figure 5 follow the Jonscher’s universal power law. Three distinct regions can be recognized throughout the whole frequency range. The frequency independent plateau region of the conductivity is in correspondence with the DC conductivity [2]. The extrapolation of plateau region to zero frequency can be used to obtain the value of DC conductivity. The DC conductivities obtained from the AC spectra (see the insets of Figure 5) are comparable with those calculated (see Table 2) from the bulk resistances given by σ_dc_ *= l/R*_b_*A*, where, *l* and *A* refer to the thickness and active area, respectively.

### 3.3. Impedance Analysis

**Figure 6:**
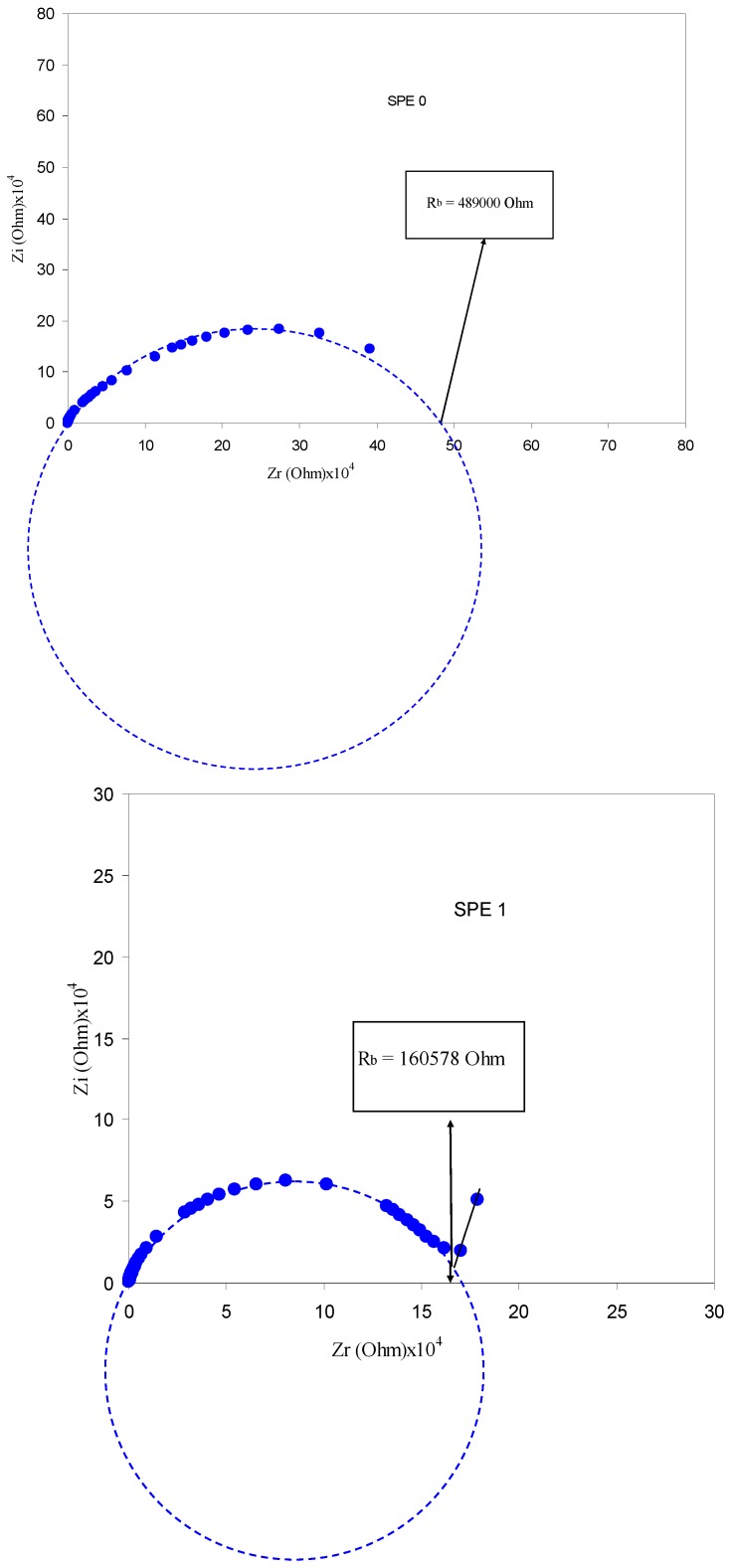

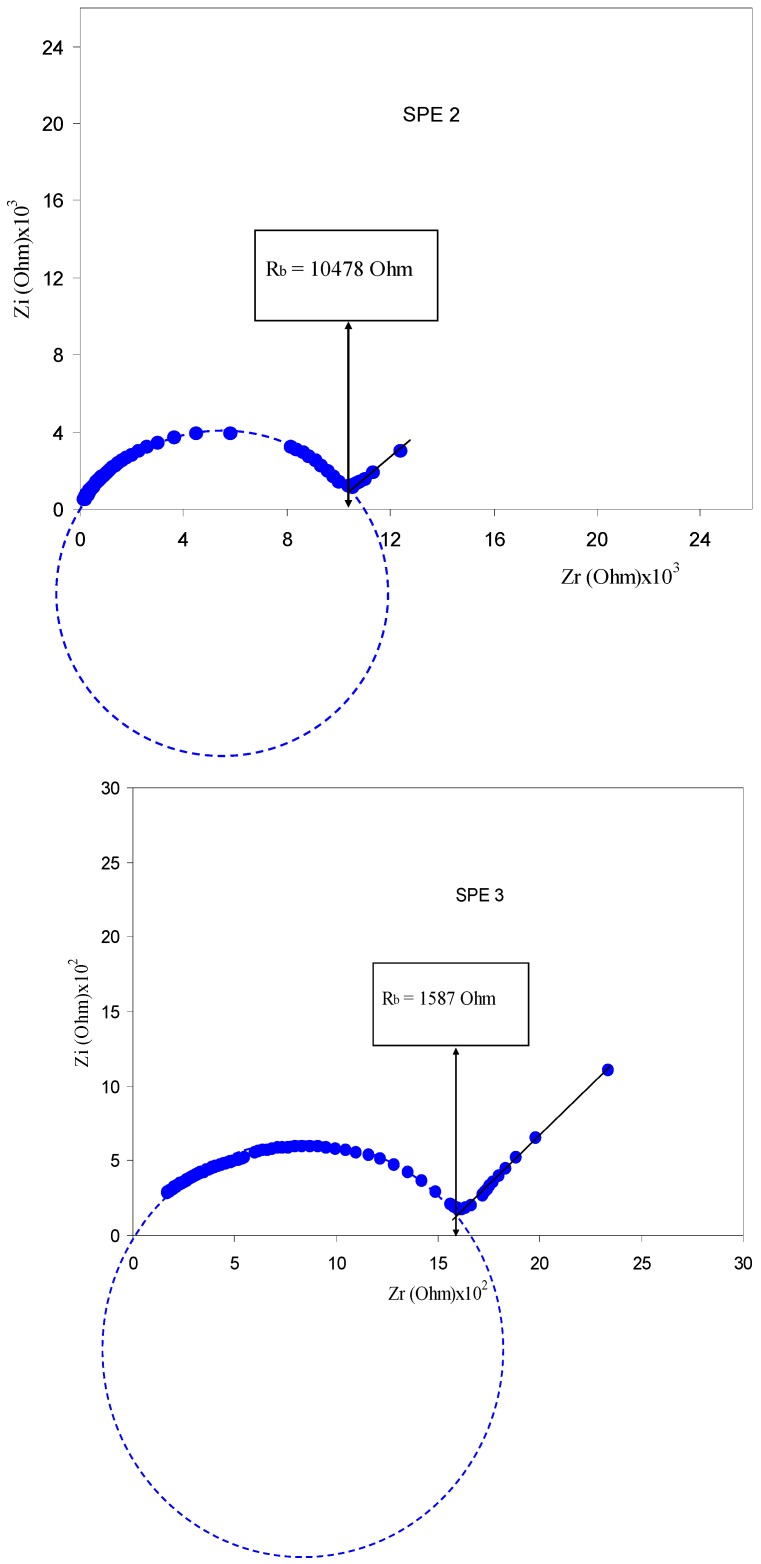

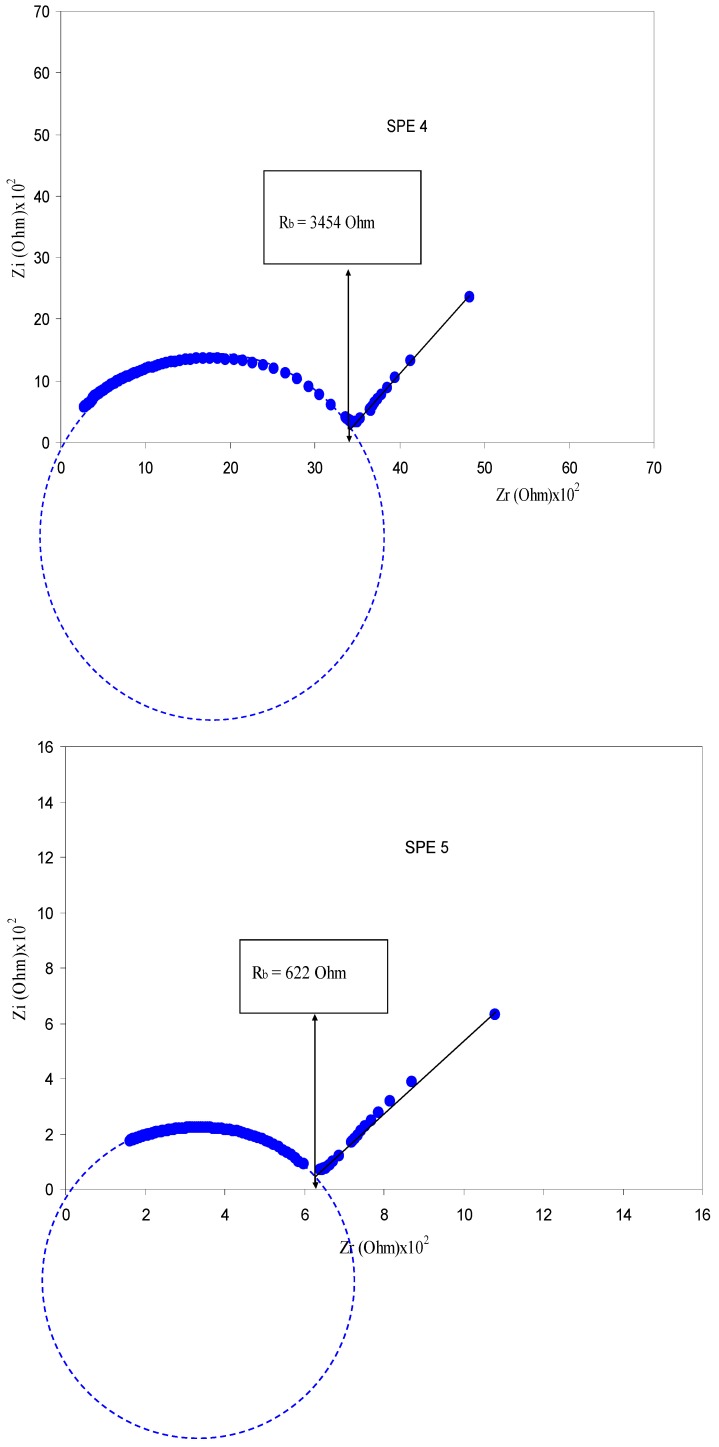
Impedance plots for pure PVA and PVA:AgNt (SPE0 to SPE5) samples. It is obvious that the spike region increases with increasing salt concentration and the high frequency semicircle decreases.

In the case of AC current, the two electrodes are charged alternately with positive and negative polarities and then an AC field produced across the polymer electrolyte, which causes the ions to move back and forth in-phase with the applied voltage. Here, the movement of ions is represented by the resistor Zr in the impedance plots, whereas the immobile polymer chains become polarized, represented by a capacitor Zi [38]. Figure 6 shows the impedance plots for all the samples at room temperature. One can see that, with increasing salt concentrations, the impedance plots exhibits an inclined straight line over the low frequency region and is attributed to the electrode polarization (EP) [39]. The ions are alternatively accumulated and depleted at each electrode as they move in the alternating field. It can be seen that on each half-cycle, ionic charges build up inside the electrolyte close to the electrodes. These charges are balanced on the electrodes by equal and opposite electronic charges. Such polarization phenomenon is called electrode polarization (EP) [38]. It is obvious that the EP region increases at high salt concentrations. The center of each semicircle was observed to be located below the real axis, which indicates that the associated relaxation processes are of non-Debye type [4,40].

### 3.4. DC Conductivity and Ion Transport Models

**Figure 7:**
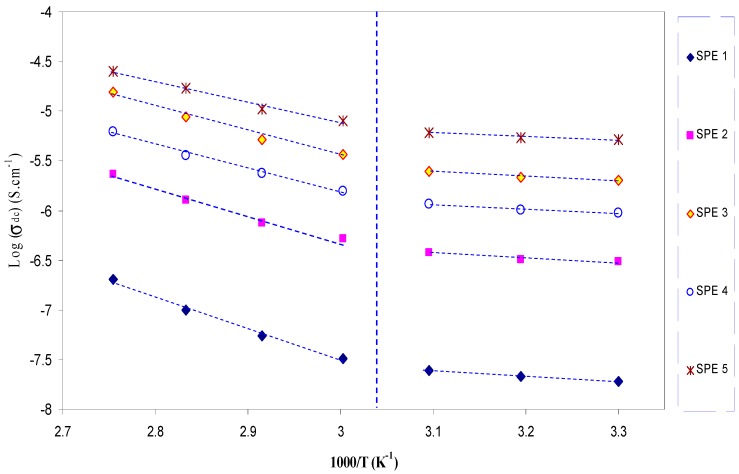
DC ionic conductivity versus 1000/T for PVA:AgNt SPE samples. The linear behavior of DC conductivity versus the reciprocal of temperature reveals that ion transport follows the Arrhenius model.

Figure 7 shows the temperature dependence of DC conductivity for all investigated doped samples. It is interesting to note that the electrical conductivity values increase with increasing temperature. Therefore, as in the case of ionic solids, the DC conductivity against 1000/T obeys the Arrhenius relationship [41],

σ_dc_ = σ_o_ exp*^[−E^*_a_*^/K^*_B_*^T]^* (2)

where *E*_a_ is activation energy and *K_B_* is the Boltzmann constant. It can be observed in Figure 7 that the temperature dependence of DC conductivity exhibits two distinguished regions. In Region I, the conductivity increases slowly with increasing temperature. However, in Region II, the conductivity increases rapidly. The activation energy calculated for SPE5 sample in the Region I was found to be lower, (*E*_a_ ≈ 0.1 eV), than the activation energy, (*E*_a_ ≈ 0.41 eV), for Region II. The activation energy may be said to indicate an energy barrier that the ion has to surmount for a successful jump between the sites [4]. As expected, the increase in temperature causes the ionic conductivity to be increased since, as temperature increases, the polymer chains flex at increased rate to create more free volume. This leads the polymer segmental mobility to be enhanced [42,43]. In Region I, the ions may transport through tunneling, while, in Region II, the ion transport may occur through hopping. The term hopping refers to the process of abrupt charge carrier displacement from one localized site to another nearby site, mainly contains both jumps over the potential barrier. This law also describes the behavior of temperature dependent conductivity within the disordered semiconductor materials [44]. An insightful study of ion conduction mechanism can be achieved from the analysis of AC conductivity at different temperatures. In our previous works, we used the behavior of frequency exponent (s) versus temperature to elucidate the temperature dependence of DC conductivity [5,36,45].

The AC conductivity has been calculated from the data of real (*Z'*) and imaginary (*Z"*) parts of the complex impedance (Z*) of the samples by using the following equation [36,45]:

(3) $${\sigma^{\prime }}_{ac}=\left[\frac{Z^{\prime }}{{Z^{\prime }}^{2}+{Z^{\prime \prime }}^{2}}\right]\times \left(\frac{t}{A}\right)$$

**Figure 8:**
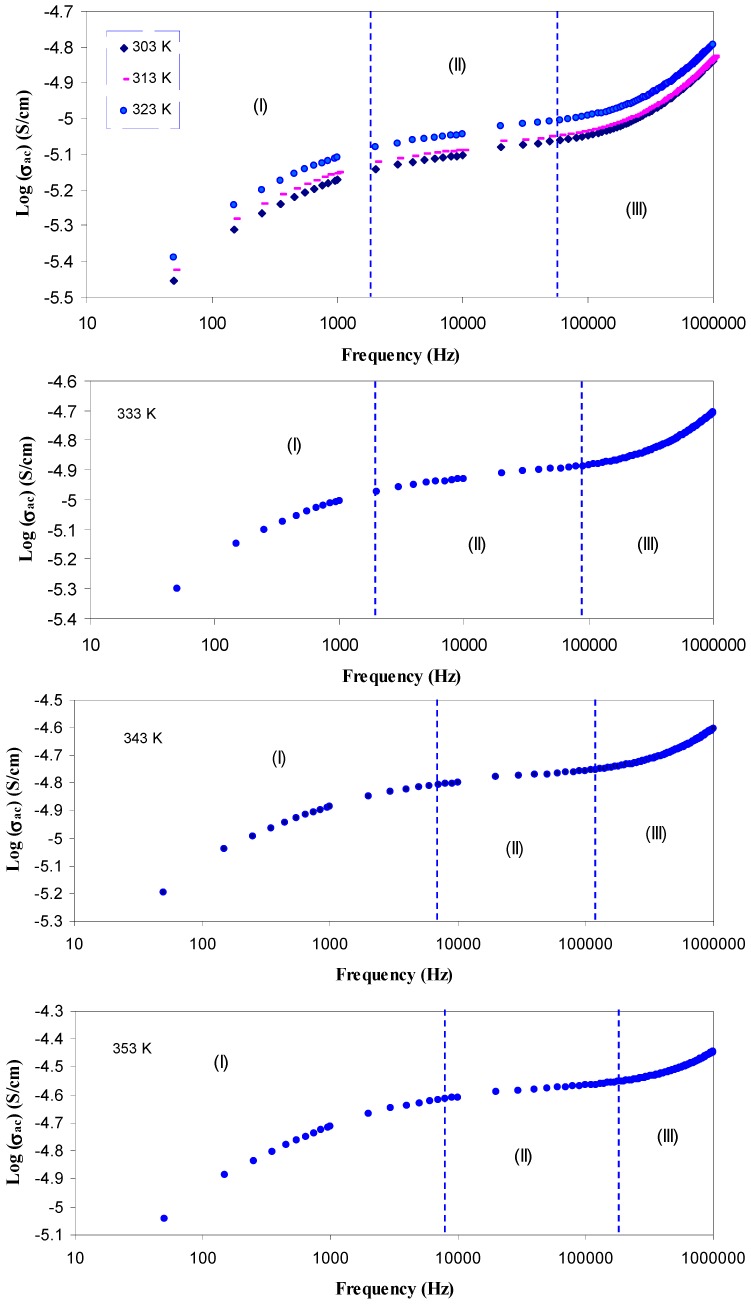

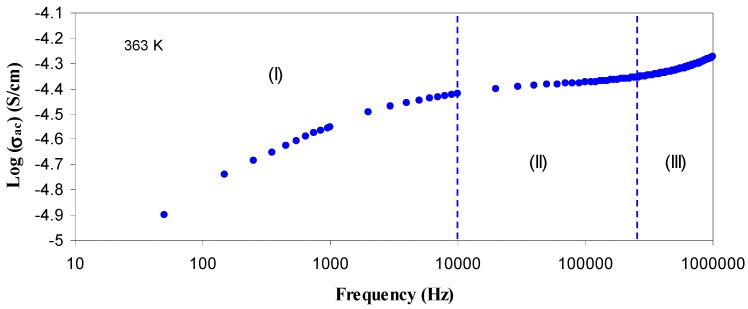
AC conductivity spectra for SPE5 sample at selected temperatures. Clearly the plateau region (Region II) shifts to higher frequency side with increasing temperature.

Figure 8 shows the AC conductivity spectra for SPE5 system at different temperatures. Here, in Figure 8, three regions can be distinguished. First, in low frequency range (Region I), there is a drop in the AC conductivity. This occurs due to the blocking ions between the electrode/electrolyte interfaces [39]. In Region II, the AC spectra are characterized by a frequency independent conductivity at different temperatures. This may be attributed to the long-range transport, that is, continual motion of mobile ions in reaction to the applied electric field, where only successful diffusion contributes to DC conductivity [40]. In Region III, the AC conductivity strongly increases with frequency and induces a wide dispersion region at temperature range between 303 and 323 K. In this temperature range, a weak temperature dependence of AC conductivity was observed indicating that ion conduction arises from tunneling through the defect sites along the polymer chains [45]. It can be seen in Figure 8 that the dispersion region is distinguishable at high temperature but shifted to higher frequency side. However, the dispersion obviously reflects a correlated motion of ions occurring on relatively short time scales (short range hopping) [46]. At high temperatures, there is a negligible interaction between the neighboring dipoles and the DC conductivity is to be dominant [47]. Therefore, at high temperature, the dispersion region decreases. The nature of ion dynamics can be determined from the well known Jonscher’s universal power law as given below [36,45]:

σ*^'^*_ac_(ω) = σ_dc_ + *A* ω*^s^* (0 < *s* < 1), (4)

**Figure 9:**
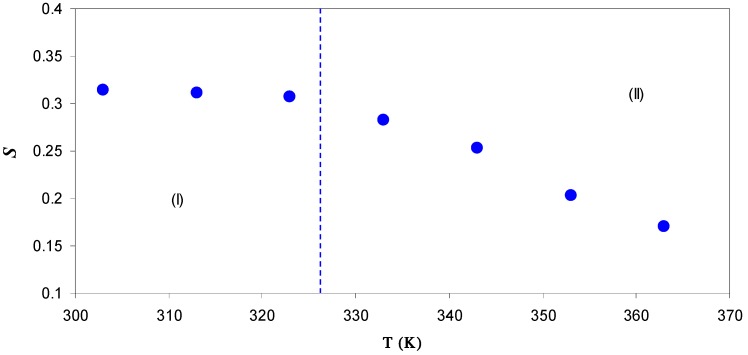
Temperature dependence of the frequency exponent (*S*) for SPE5 sample. Region I corresponds to QMT model, in which the *s* value almost temperature independent, whereas Region II reveals the dominance of CBH model.

The first term is found to be predominant for low frequencies and high temperature, while the second term is found to be predominant at high frequencies and low temperatures. The second term, which is frequency and temperature dependent term, is found to be related to the dielectric relaxation of the bound charge carriers [48]. For most ion conductors, the dispersion region of the AC conductivity is well-described by the expression, σ’_ac_(*ω*) *= A*ω*^s^*, where s is the frequency exponent and its value is generally less than unity [45]. The ion conduction mechanism can be identified based on both the frequency and temperature dependency of the AC conductivity. For the power law regions, the values of frequency exponent *s* have been estimated from the slope of plot of log (σ*'*_ac_) versus log (ω) at different temperatures. Figure 9 shows the variation of frequency exponent (*s*) as a function of temperature. It is obvious that the *S* value is almost temperature independent from 303 to 323 K, while it drops rapidly between 333 and 363 K. It is well known that the dependency of the frequency exponent (*S*) upon the temperature and frequency is a function of the conduction mechanism [49]. From the behavior of *S* value against temperature, different models have been proposed to explain the ion conduction mechanism in disordered solid electrolytes [5,36,45,48]. Among these models is a correlated barrier-hopping (CBH) model, in which, the exponent s is predicted to be frequency and temperature dependent with increasing s towards unity as T→0 K [50]. According to this model, the value of s is found to decrease with increasing temperature [51]. The temperature dependence of the frequency exponent (*S*) is observed to obey the following relation [52]:

(5) $$s\;=1-\frac{6K_{B}T}{W_{m}+\left[K_{B}T{\ln(\omega \tau }_{o})\right]}$$

**Figure 10:**
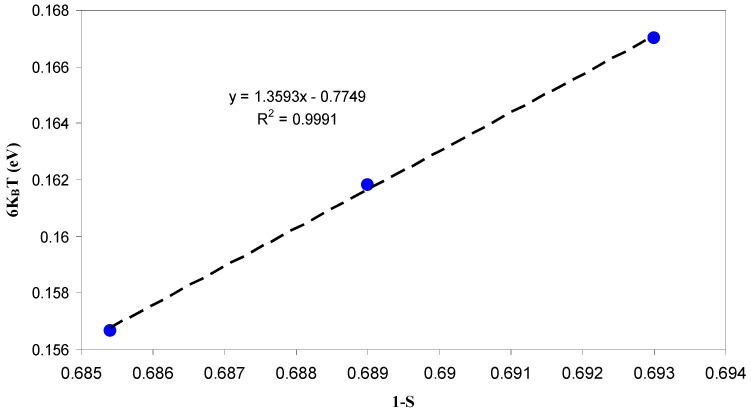
The plot of 6*K*_B_*T* versus 1−*S* or temperature ranges from 303 to 323 K. The *W*_m_ value is 1.359 eV which is higher than the activation energy (*E*_a_ ≈ 0.1 eV) for this temperature range and thus ion transport occurs through the quantum mechanical tunneling (QMT).

**Figure 11:**
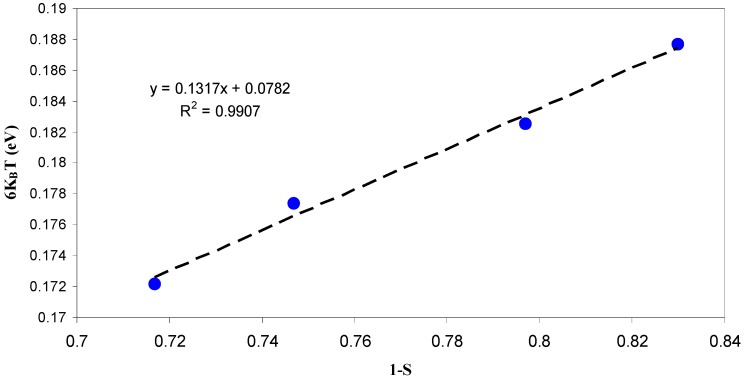
The plot of 6*K*_B_*T* versus 1−*S* for temperature ranges from 333 to 363 K. The *W*_m_ value is 0.131 eV and is lower than the activation energy (*E*_a_ ≈ 0.41 eV) for this temperature range and consequently ions transport obeys the CBH model.

It is clear in Figure 9 that the value of s decreases strongly at higher temperatures, as predicted from CBH model. Therefore, at high temperatures, an appropriate description of ion transport can be provided by the CBH model. Accordingly, the rapid increase in DC conductivity versus 1000/T (see Figure 7) can be related to the ions hopping over the barriers. The temperature independent of *S* value at low temperatures (Region I) reveals that ions are transported through tunneling. Such behavior can be explained based on quantum mechanical tunneling (QMT) model, in which the temperature independent of the frequency exponent (*S*) is revealed [45]. Therefore, the weak temperature dependent-behavior of the DC conductivity shown in Figure 7 can also be interpreted using the QMT model. Deep understanding of the mechanism of ionic conduction can be obtained from the comparison of *E*_a_ values with the *W*_m_ values. The plots of *6K*_B_*T* versus (1−*S*) were used to estimate the *W*_m_ values. The maximum barrier height (*W*_m_) for Region I of Figure 9 is found to be 1.35 eV (see inset of Figure 10), which is higher than the activation energy (0.1 eV). This means that the ions conduction mechanism cannot be occurred through the hopping transport but they must transport via tunneling. On the other hand, the maximum barrier height (*W*_m_) for Region II of Figure 9 is found to be 0.131 eV (see inset of Figure 11), which is lower than the activation energy (0.41 eV). Consequently, ions conduction mechanism occurs via the hopping transport. To our knowledge, this is the first paper that makes a correlation between the activation energy and barrier heights to shed light on ion transport mechanism. From the DC and AC conductivity studies presented in this work, it is understood that ion transport mechanism is a complicated issue in solid polymer electrolytes. 

## 4. Conclusions

In this paper, the role of ion dissociation on silver nanoparticle formation and DC conductivity in PVA:AgNO_3_ based solid polymer electrolyte has been studied. The silver-ion conducting solid polymer electrolyte samples have been prepared by solution cast technique. The technique of UV–Vis absorption spectrum has been used to investigate the formation of silver nanoparticles. Broad and sharp peaks due to surface plasmonic resonance (SPR) of silver nanoparticles have been observed. For the sample with high dielectric constant, maximum SPR peak intensity has been exhibited. From impedance plots, the diameter of high frequency semicircle was found to be dependent on the conductivity of the samples. The correlation between DC conductivity and dielectric constant has been explained. The DC conductivity obtained from the AC conductivity spectra was almost close to that calculated from the bulk resistance. The temperature dependence of the DC conductivity was found to obey the Arrhenius equation within two distinguishable regions. The AC conductivity has been studied at different temperatures in order to understand the ion conduction mechanism. The AC conductivity versus frequency was found to follow Jonschers universal power law. Three distinct regions were recognized in the AC spectra. The frequency exponent (*s*) was calculated for the dispersion regions of AC conductivity. The behavior of *s* value with temperature was used to interpret the pattern of DC conductivity versus 1000/T. Deep insights on ion transport mechanism were understood from the calculated activation energy (*E*_a_) and maximum barrier height (*W*_m_). Here, the achieved results revealed that ion transport occurred by tunneling, i.e., followed QMT model, when the barrier height was higher than the activation energy. However, when the activation energy was higher than the barrier heights, ion transport occurred by hopping, and obeyed the CBH model.

## Figures and Tables

**Table 1:** The composition of PVA:AgNt based solid polymer electrolytes.

Sample Designation	PVA (g)	AgNt (wt %)	AgNt (g)
SPE0	1	0	0.0000
SPE1	1	5	0.0526
SPE2	1	10	0.1111
SPE3	1	15	0.1765
SPE4	1	20	0.2500
SPE5	1	25	0.3333

**Table 2:** DC ionic conductivity and ε′ of pure PVA and PVA:AgNt complexes at ambient temperature.

Sample Designation	DC Conductivity (S/cm)	Dielectric Constant
SPE0	6.48 × 10^−9^	3.1
SPE1	2.1 × 10^−8^	3.9
SPE2	3.06 × 10^−7^	6.27
SPE3	2.3 × 10^−6^	9.2
SPE4	1.01 × 10^−6^	4.97
SPE5	4.5 × 10^−6^	10.8
